# Prescribed but not approved: biologic agents used without approval in juvenile idiopathic arthritis in Switzerland, France and Belgium

**DOI:** 10.1186/1546-0096-12-S1-P338

**Published:** 2014-09-17

**Authors:** Andreas Woerner, Alexandre Belot, Etienne Merlin, Carine Wouters, Gerald Berthet, Anuela Kondi, Daniela Kaiser, Laetitia Higel, Anne Maes, Elvira Cannizzaro, Natalia Cabrera, Silke Schroeder, Florence Aeschlimann, Annette von Scheven, Agnès Duquesne, Samuel Roethlisberger, Isabelle Kone-Paut, Michael Hofer

**Affiliations:** 1 Pediatric Rheumatology, University Children`s Hospital, Basel, Switzerland; 2 Rhumatologie pédiatrique Romande, Centre Hospitalier Universitaire Vaudois, Lausanne, Switzerland; 3 Pediatric Rheumatology, Hôpital Femme Mère Enfant, Lyon, France; 4 Pediatrics, Centre Hospitalier Universitaire, Clermont-Ferrand, France; 5 Pediatric Rheumatology, University Hospital, Leuven, Belgium; 6 Pediatrics, Children`s Hospital, Aarau, Switzerland; 7 Pediatrics, Kremlin-Bicêtre Hospital, Paris, France; 8 Pediatrics, Children`s Hospital, Lucerne, Switzerland; 9 Pediatrics, Hôpital Hautepierre, Strasbourg, France; 10 Pediatric Rheumatology, University Children`s Hospital, Zuerich, Switzerland; 11 Pediatric Rheumatology, Kremlin-Bicêtre Hospital, Paris, France

## Introduction

Biologic agents (BA) have profoundly changed the outcome of juvenile idiopathic arthritis (JIA), making inactive disease and clinical remission an achievable goal for treatment. An increasing number of BA has become available in the last 15 years. However, some BA that have been associated to efficacy in some clinical conditions are not approved by legal authority for the use in pediatric population.

## Objectives

To evaluate the frequency of pediatric patients with JIA treated with BA not approved by medical supervisory authorities in Switzerland, France and Belgium at initiation of therapy.

## Methods

Multicenter, retrospective study using the juvenile inflammatory rheumatism (JIR) cohort, including ten Swiss, French and Belgian centers for pediatric rheumatology.

## Results

A total of 796 BA treatments in 531 patients were collected. Mean age at start of first biologic therapy was 10.9 (SD ± 4.61) years. Etanercept, the first approved BA for pediatric use, was initiated in 378 patients (47.5%), of whom 377 (99.7%) after the approval date of the European medical agency (EMA) or Swissmedic (SM). Adalimumab, infliximab and golimumab were used in 147 (18.5%), 106 (13.3%) and 14 (1.8%) patients, respectively; 75 (51.0%) patients were started on adalimumab before EMA/SM approval, whereas all patients on infliximab and golimumab were treated without EMA/SM approval. Abatacept was given in 26 patients (3.3%), of whom in 10 patients (38.5%) before EMA/SM approval. Tocilizumab was used in a total of 48 patients (6.0%); for systemic-onset JIA and non-systemic JIA, it was prescribed in 5 of 28 patients (17.9%) and 16 of 20 (80%) before EMA/SM approval, respectively. Canakinumab used for the treatment of systemic-onset JIA was given in 14 patients (77.8%) without approval for this indication. Anakinra was identified in 49 patients (6.3%) for the treatment of systemic-onset JIA, although EMA/SM approval is pending for this disease. When more than one BA was used in a patient, 167 out of 265 treatments (63%) were given without approval. In total, 300 treatments (37.7%) were started without authorization by EMA/SM.

## Conclusion

In pediatric rheumatology clinical practice, a significant number of BA lacks authority approval for the treatment of JIA. Pediatric clinical trials and registers are crucial to assess effectiveness and safety of BA in this rare disease, substantiating an unequivocal decision making of both doctors and their patients.

## Disclosure of interest

None declared

